# Constant Light Dysregulates Cochlear Circadian Clock and Exacerbates Noise-Induced Hearing Loss

**DOI:** 10.3390/ijms21207535

**Published:** 2020-10-13

**Authors:** Chao-Hui Yang, Chung-Feng Hwang, Jiin-Haur Chuang, Wei-Shiung Lian, Feng-Sheng Wang, Ethan I. Huang, Ming-Yu Yang

**Affiliations:** 1 Department of Otolaryngology, Kaohsiung Chang Gung Memorial Hospital and Chang Gung University College of Medicine, Kaohsiung 83301, Taiwan; chouwhay@gmail.com; 2 Graduate Institute of Clinical Medical Sciences, College of Medicine, Chang Gung University, Tao-Yuan 33302, Taiwan; jhchuang@adm.cgmh.org.tw (J.-H.C.); wangfs@ms33.hinet.net (F.-S.W.); 3 Division of Pediatric Surgery, Kaohsiung Chang Gung Memorial Hospital and Chang Gung University College of Medicine, Kaohsiung 83301, Taiwan; 4 Core Laboratory for Phenomics & Diagnostics, Department of Medical Research, Kaohsiung Chang Gung Memorial Hospital and Chang Gung University College of Medicine, Kaohsiung 83301, Taiwan; lianws@gmail.com; 5 Department of Otolaryngology, Chang Gung Memorial Hospital, Chiayi 61363, Taiwan; ehuang@alumni.pitt.edu

**Keywords:** circadian dysregulation, clock genes, noise-induced hearing loss, sensory hair cells, synaptic ribbons

## Abstract

Noise-induced hearing loss is one of the major causes of acquired sensorineural hearing loss in modern society. While people with excessive exposure to noise are frequently the population with a lifestyle of irregular circadian rhythms, the effects of circadian dysregulation on the auditory system are still little known. Here, we disturbed the circadian clock in the cochlea of male CBA/CaJ mice by constant light (LL) or constant dark. LL significantly repressed circadian rhythmicity of circadian clock genes *Per1*, *Per2*, *Rev-erbα*, *Bmal1*, and *Clock* in the cochlea, whereas the auditory brainstem response thresholds were unaffected. After exposure to low-intensity (92 dB) noise, mice under LL condition initially showed similar temporary threshold shifts to mice under normal light–dark cycle, and mice under both conditions returned to normal thresholds after 3 weeks. However, LL augmented high-intensity (106 dB) noise-induced permanent threshold shifts, particularly at 32 kHz. The loss of outer hair cells (OHCs) and the reduction of synaptic ribbons were also higher in mice under LL after noise exposure. Additionally, LL enhanced high-intensity noise-induced 4-hydroxynonenal in the OHCs. Our findings convey new insight into the deleterious effect of an irregular biological clock on the auditory system.

## 1. Introduction

The circadian clock and rhythm are important for the regulation of tissue homeostasis and function. Expanding evidence has revealed that daily rhythmic changes modulate a plethora of physiological activities, like sleep, appetite, and hormone production, affecting metabolism and gene expression in various tissues [1]. The suprachiasmatic nucleus (SCN) of the anterior hypothalamus plays an important role in maintaining the internal clock to control physiological activities in a regular 24 h cycle. Increasing reports have uncovered that circadian oscillators are also present in peripheral tissues, including liver, heart, kidney, and peripheral blood leukocytes [2]. More than nine core circadian clock genes control circadian oscillation through the transcriptional and translational feedback loop [1,3,4], and sustain the circadian rhythm of mammalian central and peripheral tissues [5].

Although circadian oscillation is found in several peripheral tissues of the human body, circadian regulation in the auditory system is only beginning to be understood from animal models. Earlier studies indicated that rats living in a constant dark condition showed a poor acoustic startle response [6,7]. The severity of kanamycin-mediated ototoxicity is correlated with diurnal sensitivity of rats [8]. Recently, it was reported that mice receiving noise exposure at night suffered permanent hearing loss while the same exposure during the day resulted in temporary hearing loss only [9]. Park et al. demonstrated a circadian clock in the cochlea and inferior colliculus of adult mice [10] as well as a differential phase arrangement of cellular clocks along the tonotopic axis (base to apex) of mouse cochlear explants [11]. These findings imply a possible role of circadian regulation in auditory function [12,13].

In modern society, social activities and working demands increase the chances of humans staying active at night. Changing a diurnal lifestyle to a nocturnal one prolongs wakefulness in the night and disturbs the circadian rhythm throughout the body. Circadian disruption leads to the interruption of physiological oscillation networks and ultimately escalates tissue dysfunction that increases the risk of disease development [14]. Disrupted circadian rhythm or dysregulated circadian clock genes have been correlated with the development of depression [15], diabetes [16], cancer [17], cardiovascular diseases [18], and degenerative neurologic disorders [19,20]. We previously revealed that alteration of circadian clock genes was present in patients with sudden sensorineural hearing loss [21]. In addition to the irregular circadian lifestyle in modern humans, excess sound in workplaces and entertainment also increases the risk of noise overexposure, which makes noise-induced hearing loss one of the most common causes of acquired hearing loss today [22]. Since people with a lifestyle of irregular circadian rhythms (such as factory shift workers and musicians in clubs) are frequently also populations with excessive exposure to noise, it would be interesting to investigate if circadian dysregulation could impair cochlear function or affect the consequences of noise exposure.

This study aimed to utilize male CBA/CaJ mice in constant light (LL) or constant dark (DD) conditions to dysregulate the circadian clock in the cochlea. We then investigated the impact of circadian dysregulation on auditory thresholds and cochlea after low- or high-intensity noise exposure.

## 2. Results

### 2.1. Circadian Oscillation Was Present in the Cochlea

**Figure 1:**
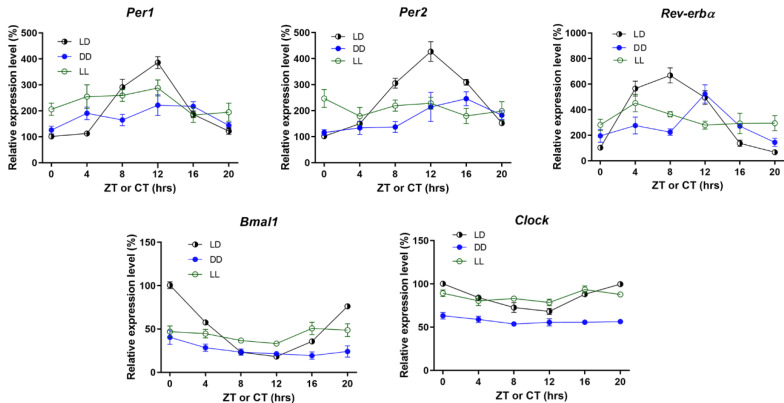
Relative transcript profiles of circadian clock genes in the cochlea at 6 zeitgeber times (ZTs) in the LD (light–dark), and at 6 circadian times (CTs) in constant dark (DD) and constant light (LL) groups. Each value represents the percentage change relative to ZT0 of the LD group, which was normalized to 100%. Data are mean ± SEM. *n* = 8–10 for each time point in each condition.

We first characterized circadian oscillation profiles in the cochlea of mice living in a normal light–dark (LD) cycle (12 h/12 h LD cycle with the light intensity kept at about 270–320 lux (lm/m^2^) during light-on and darkness during light-off). Cochlear mRNA expression of circadian clock genes was analyzed at six different ZTs (zeitgeber times) every 4 h. Circadian expression of *Per1*, *Per2*, and *Rev-erbα* were present through the study, whereas *Bmal1* and *Clock* expression was changed in antiphase circadian fashion (Figure 1 and Table 1, LD group). In the cochlea, the localization of PER2 was mainly observed in the organ of Corti, with strong PER2 immunofluorescence at zeitgeber time 12 (ZT12) and weak immunoreaction at ZT4 (Supplementary Figure S1). These findings confirmed the presence of circadian oscillation in the cochlea.

### 2.2. LL Dysregulated Cochlear Circadian Oscillation

To evaluate whether constant light (LL) or constant dark (DD) could affect the circadian rhythm in the cochlea, we checked the circadian clock genes’ mRNA transcripts in the cochlea of mice under LL or DD condition for 4 weeks (Figure 1). In the DD group, loss of circadian rhythm was evident in *Bmal1* and *Clock*, whereas circadian oscillations were still present in *Per1*, *Per2*, and *Rev-erbα*. In contrast, the circadian expression of *Per1*, *Per2*, *Rev-erbα*, *Bmal1*, and *Clock* were all dampened under the LL condition (Table 1). These results showed that LL disrupted circadian oscillation more in the cochlea. Therefore, LL was used as the model for succeeding experiments to test the impact of circadian dysregulation on the cochlear physiology and the effects of noise exposure.

### 2.3. LL Did Not Affect Baseline Auditory Thresholds and Neural Response Amplitudes

**Figure 2:**
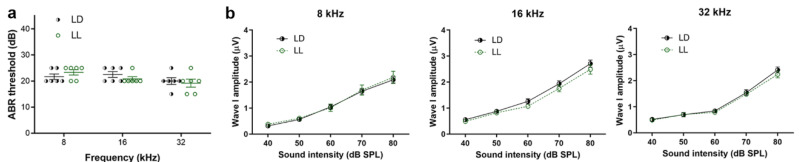
The ABR (auditory brainstem response) thresholds and wave I amplitudes in the LD and LL groups. (**a**). The ABR thresholds in the LD and LL groups. (**b**). Wave I amplitudes at sound intensities of 40 to 80 dB in the LD and LL groups at 8, 16, and 32 kHz. There were no significant differences in ABR thresholds and wave I amplitudes between the LD and LL groups. Data are mean ± SEM (*n* = 6 for each group).

We examined if LL condition affected auditory thresholds of mice, which were detected by auditory brainstem responses (ABRs). In normal CBA/CaJ mice, the baseline auditory thresholds were less than 25 dB SPL. Mean ABR thresholds after the 4-week LL were around 20 dB SPL, which were not significantly different from the LD group at 8, 16 and 32 kHz (Figure 2a). Likewise, wave I amplitudes, which reflected neural responses of the auditory nerve, were similar at sound intensities of 40 to 80 dB stimulus between LD and LL groups (Figure 2b). The results suggest that LL for 4 weeks did not affect baseline cochlear thresholds and auditory nerve activity.

### 2.4. LL Did Not Affect Low-Intensity Noise-Induced Temporary Threshold Shift (TTS) and ABR Wave I Amplitudes Changes

**Figure 3:**
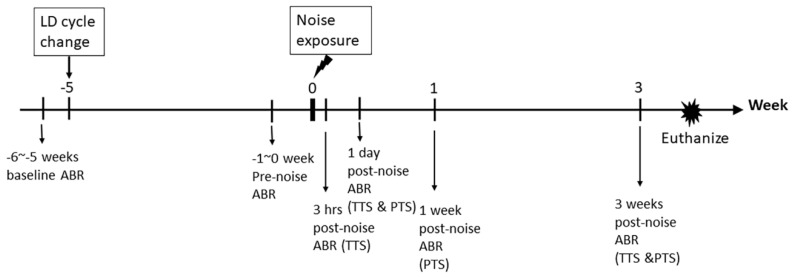
Timeline of experiments for testing the auditory brainstem response (ABR). The ABRs were tested before normal light–dark cycle change, and before noise exposure. Post-noise ABRs were tested at 3 h, 1 day, and 3 weeks after noise exposure in the TTS (temporary threshold shift) experiments, and at 1 day, 1 week, and 3 weeks after noise exposure in the PTS (permanent threshold shift) experiments.

**Figure 4:**
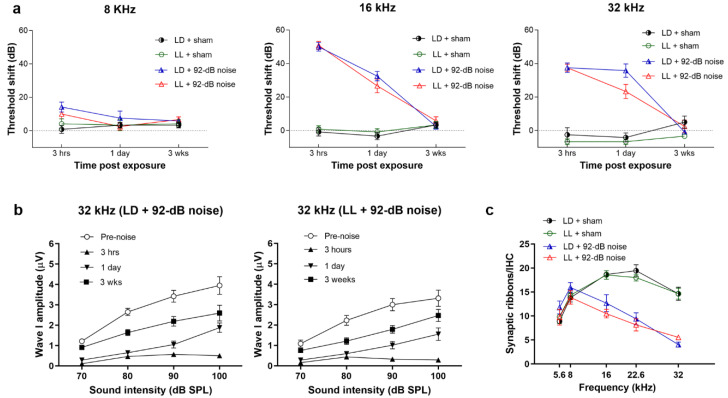
The ABR threshold shifts, wave I amplitudes, and synaptic ribbon counts in the LD and LL groups after 92 dB noise exposure. (**a**) No significant differences in the threshold shifts were observed between the LD and LL groups at 8, 16, and 32 kHz after the 92 dB noise exposure. Data are mean ± SEM (*n* = 6 for each group). (**b**) ABR wave I amplitudes at 32 kHz were mostly attenuated at 3 h after the 92 dB noise exposure and increased later both in the LD and LL groups. There were no differences in wave I amplitudes between LD + 92 dB and LL + 92 dB groups. (**c**) A decreased number of synaptic ribbons were found over 16, 22.6, and 32 kHz at 3 weeks after the 92 dB noise. No significant difference in synaptic ribbon counts was observed between the LD and LL groups postexposure. Data are mean ± SEM (*n* = 5 for each group).

To investigate whether LL affected the auditory thresholds during noise-induced hearing loss, we used two models, low-intensity noise (92 dB) or high-intensity noise (106 dB), to induce temporary or permanent threshold shift in CBA/CaJ mice respectively [23] (Figure 3). Animals exposed to no noise were designated in the sham group. In the LD group, 92 dB noise resulted in threshold shifts at 3 h after noise exposure and returned to baseline 3 weeks later, which represented TTS (temporary threshold shift) (Figure 4a). Likewise, exposure to 92 dB noise in the LL group also caused similar threshold shifts initially, and finally returned to baseline thresholds. These results indicated that low-intensity noise in both the LD and LL group caused TTS only.

Since decreased ABR wave I amplitudes and loss of synaptic ribbons (cochlear synaptopathy) persist for several weeks at high frequency after low-intensity noise even while ABR has recovered [24], we measured ABR wave I amplitudes over 32 kHz at 3 h, 1 week and 3 weeks after 92 dB noise exposure. Wave I amplitudes decreased significantly at 32 kHz by 3 h after noise exposure and recovered to about 60% 3 weeks later both in the LD and LL groups, while the differences between the two groups were not significant (Figure 4b). The densities of synaptic ribbons per IHC (inner hair cell) were decreased in the noise groups at three weeks after 92 dB noise over 16, 22.6, and 32 kHz, while synaptic ribbon counts were similar in LL and LD groups without significant difference (*p* = 0.315) (Figure 4c). These results suggested that LL did not affect low-intensity noise-induced synaptopathy.

### 2.5. LL Augmented High-Intensity Noise-Induced Permanent Threshold Shift (PTS) and Outer Hair Cell (OHC) Loss

**Figure 5:**
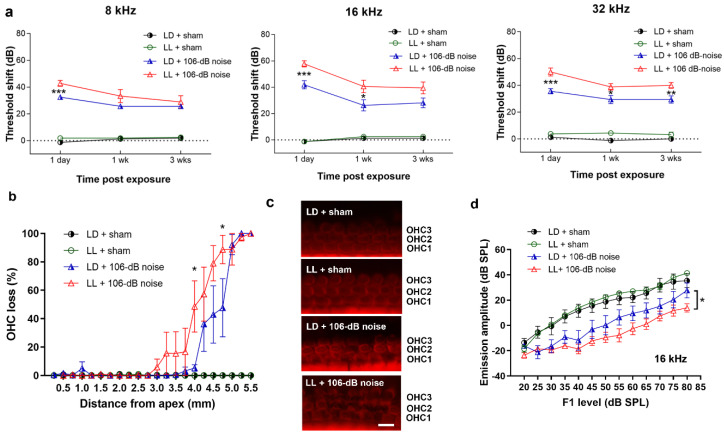
The ABR threshold shifts, OHC (outer hair cell) counts, and DPOAE 2F1-F2 amplitudes in the LD and LL groups after 106 dB noise exposure. (**a**) Significantly higher threshold shifts in the LL group were found over 8 kHz at 1 day postexposure, over 16 kHz at 1 day and 1 week postexposure, and over 32 kHz at 1 day, 1 week, and 3 weeks postexposure. Data are mean ± SEM (*n* = 8–9 for each group). * *p* < 0.05, ** *p* < 0.01, *** *p* < 0.001 for LD + 106 dB noise vs. LL + 106 dB noise. (**b**) The LL group showed a higher percentage of outer hair cell (OHC) loss in the basal segment than the LD group after exposure to the 106 dB noise. Data are mean ± SEM (LD + sham, *n* = 7; LL + sham, *n* = 6; LD + 106 dB noise, *n* = 5; LL+ 106 dB noise, *n* = 6). * *p* < 0.05 for LD + 106 dB noise vs. LL + 106 dB noise. (**c**). Representative images of OHCs in the basal segment (about 4 mm from apex) after different treatments. OHC 1, 2, and 3 indicate three rows of OHCs. Scale bar = 10 µm (**d**) DPOAE 2F1-F2 amplitudes were decreased in the LL group after exposure to the 106 dB noise at 16 kHz. Data are mean ± SEM. * *p* < 0.05 for LL + 106 dB noise vs. LD + 106 dB.

We next investigated whether LL affected ABR thresholds shift after high-intensity (106 dB) noise exposure. In contrast to the sham groups, threshold shifts in 106 dB noise groups persisted for up to 3 weeks at all tested frequencies of 8, 16, and 32 kHz, which showed PTS (permanent threshold shift) (Figure 5a). Compared to the LD group, the LL group had significantly higher threshold shifts over 8 kHz (*p* < 0.001), 16 kHz (*p* < 0.001), and 32 kHz (*p* < 0.001) at one day after 106 dB noise exposure. The significantly increased thresholds shift in the LL group persisted up to 3 weeks after exposure over 32 kHz (*p* = 0.005). These results revealed that LL augmented high-intensity noise-induced PTS, particularly over high frequency.

Loss of OHCs (outer hair cells) is one of the major cochlear pathologies in permanent noise-induced hearing loss [23]. We therefore checked OHCs at 3 weeks after a 106 dB exposure in cochlear surface preparations. OHC loss was present in a base-to-apex progression in the noise-exposed groups. LL resulted in significant increases in OHC death over basal segment at 4 mm (*p* = 0.02) and 4.75 mm (*p* = 0.046) from the apex (Figure 5b,c). We further investigated the OHC function at 16 kHz, the region without OHC loss (about 2.83 mm from the apex) by distortion product otoacoustic emissions (DPOAEs). DPOAE 2F1-F2 emission amplitudes were significantly different between the four groups in the 16 kHz-region (*p* < 0.001), while post-hoc test revealed that noise exposure significantly reduced DPOAE amplitudes in the LL group (*p* = 0.02) (Figure 5d). These results demonstrate that LL renders OHC vulnerable to high-intensity noise.

### 2.6. LL Increased High-Intensity Noise-Induced Reduction of Synaptic Ribbons

**Figure 6:**
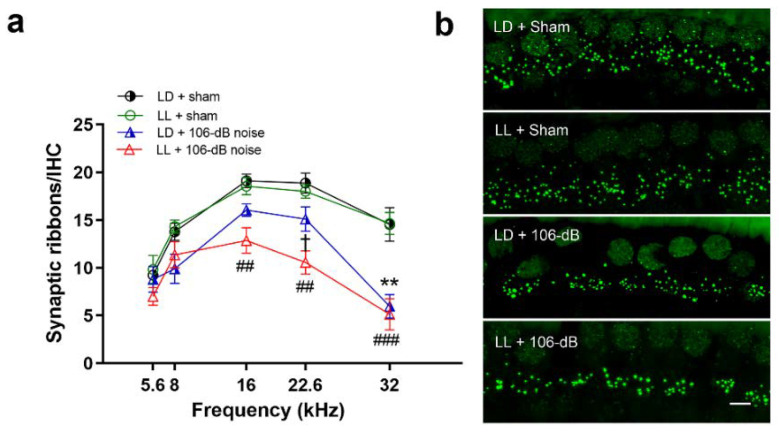
Synaptic ribbon counts in the LD and LL groups after 106 dB noise exposure. (**a**) The number of synaptic ribbons decreased significantly in the LD group at 32 kHz and the LL group at 16, 22.6, and 32 kHz after 106 dB noise exposure, while the significant difference between LD and LL groups was observed at 22.6 kHz. Data are mean ± SEM (*n* = 5–6 for each group). ** *p* < 0.01 for LD + 106 dB noise vs. LD + sham, ## *p* < 0.01, ### *p* < 0.001 for LL + 106 dB noise vs. LL + sham, † *p* < 0.05 for LD + 106 dB noise vs. LL + 106 dB noise. (**b**) Representative images of synaptic ribbons stained by CTBP2 (green dots) over the 22.6 kHz region. Scale bar = 5 µm.

Since cochlear synaptopathy can also occur after high-intensity noise exposure [25], we further checked the synaptic ribbons over inner hair cells (IHCs) at 3 weeks after exposure. High-intensity noise significantly reduced synaptic ribbons at 16 kHz (*p* < 0.001), 22.6 kHz (*p* < 0.001), and 32 kHz (*p* < 0.001) regions (Figure 6a). Further analysis showed that noise exposure significantly reduced the synaptic ribbon in the LD group at 32 kHz (*p* = 0.005) and LL group at 16 (*p* = 0.003), 22.6 (*p* = 0.001) and 32 kHz (*p* < 0.001). LL increased more high-intensity noise-induced reduction of synaptic ribbons, particularly over 22.6 kHz (*p* = 0.04) (Figure 6a,b). This result suggested that LL augmented cochlear synaptopathy by high-intensity noise.

### 2.7. LL Increased High-Intensity Noise-Induced 4-Hydroxynonenal (4-HNE) in OHCs

**Figure 7:**
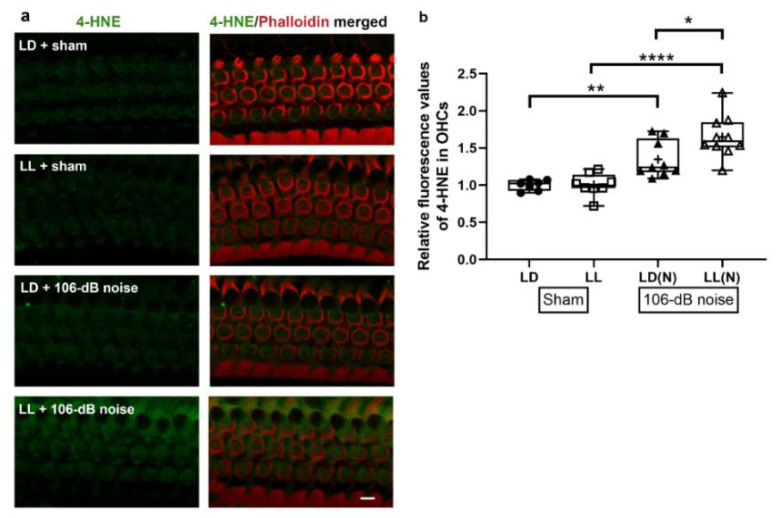
4-HNE immunolabeling in cochlear surface preparations. (**a**) Representative images of immunoreactivity for 4-HNE in different treatments. Scale bar = 5 µm. (**b**) Quantification of 4-HNE fluorescence in OHCs revealed significantly increased 4-HNE in LD and LL groups after 106 dB noise exposure, while stronger immunolabeling for 4-HNE was observed in the LL(N) group compared to the LD(N) group. Boxplots indicate median and interquartile. + indicates mean value. * *p* < 0.05, ** *p* < 0.01, **** *p* < 0.0001.

The markers of oxidative stress, such as 4-HNE (the product of lipid oxidation), increase in OHCs after high-intensity noise exposure [23,26]. So, we further evaluated the 4-HNE levels in the OHCs at 1 h after 106 dB noise in cochlear surface preparations (Figure 7a). Immunoreactivity for 4-HNE was significantly different between the four groups in OHCs (*p* < 0.001). Post-hoc quantification analysis of 4-HNE immunolabeling revealed that 106 dB noise significantly increased 4-HNE in the LD (*p* = 0.02) and LL (*p* < 0.001) groups, while a significantly stronger immunoreactivity for 4-HNE was observed in the LL group compared to the LD group after noise exposure (*p* = 0.02) (Figure 7b). These results suggest that high-intensity noise augmented more oxidative stress in OHCs of the LL mice.

## 3. Discussion

In agreement with the groundbreaking studies of Canlon and colleagues [9], we demonstrated the oscillated expression of circadian clock genes in the cochlea of CBA/CaJ mice. We further showed that cochlear circadian dysregulation by LL is detrimental to the inner ear, causing greater damage of hair cells and synaptic ribbons during high-intensity noise exposure. The effects were most predominant over the higher frequency region of the cochlea, the region mainly affected in noise-induced hearing loss. Our results clarify that the dysregulation of the cochlear circadian clock can influence auditory function and sensitivity of the inner ear during acoustic trauma.

Light is the most important zeitgeber and central modulator for circadian clocks [27]. The manipulation of environmental light has frequently been used to test the effect of circadian disruption on the physiology and behavior of animals [28,29,30] and prolonged exposure to LL or DD is one of the common models [30,31,32,33,34,35]. We demonstrate here that exposure of mice to LL resulted in the loss of rhythms in five cochlear circadian clock genes, which is in line with previous studies showing a dampened amplitude of circadian clock gene expression under LL condition in the SCN [36] and peripheral clocks [35].

Change of the LD cycle also impacts the complexity of neurons and behavior in mice [29,37]. Therefore, we tested the cochlear physiology and pathophysiology of mice undergoing circadian dysregulation by prolonged exposure to LL. There were no significant differences in ABR thresholds or neural response amplitudes between LL and LD groups. Even with the severe dampening of cochlear circadian rhythm by LL, the cochlear physiology seems normal. If any alterations of neurotransmitter signaling [38] or desynchronization of neuronal population [37] occur after LL, they are not sufficient to affect threshold sensitivity.

In view of the diurnal sensitivity to noise trauma [9], we further hypothesized that circadian dysregulation by LL may affect the recovery of cochlear threshold shifts in mice following noise exposure. Surprisingly, the ABR thresholds in the LL group completely recovered to pre-exposure levels at three weeks after a low-intensity noise exposure. Moreover, acute cochlear nerve denervation in the LL group, demonstrated by the reduction of suprathreshold wave I amplitude [24], recovered to a similar extent three weeks later in the LD group, suggesting similar decrements of synaptic ribbons in both groups. In contrast, LL augmented high-intensity noise-induced PTS, which is characterized by the death of cochlear sensory cells. These results suggested that circadian dysregulation has different impacts on the pathologic processes underlying temporary and permanent threshold shift [39,40].

As a consequence, the link of LL-induced circadian dysregulation to hair cell pathology in PTS may be explained by the featured molecular oscillators in the neurosensory damage after high-intensity noise. A well-documented hypothesis of traumatic permanent noise-induced hearing loss is oxidative stress in OHCs, characterized by excess reactive oxygen species (ROS) production induced by intense noise exposure [26,41] overwhelming the intrinsic antioxidant defense system. Previous literature has demonstrated the rhythmicity of the cellular antioxidant system, including superoxide dismutase, peroxiredoxin, and glutathione [42,43], which are also important defense systems in hair cells in response to acoustic insults [44,45]. In the cochlea, we observed that PER2 protein was most expressed in the organ of Corti (Supplementary Figure S1a), so the presence of circadian PER2 and antioxidant oscillation in OHCs is possible. Additionally, LL decreased amplitudes of cochlear circadian clock gene mRNA expression and increased 4-HNE in OHCs in response to noise, which is in line with a previous publication showing that LL influences the circadian oscillation of antioxidants [46] and induces oxidative stress [47]. Although the detailed mechanisms need to be investigated further, it is likely that LL attenuated the amplitude of antioxidant oscillation in OHCs, which resulted in increased noise vulnerability. The circadian effects of LL condition on all the longitudinal regions of cochlear sensory epithelium can be revealed by the elevated ABR threshold shifts at one day after 106 dB noise exposure at 8 k, 16 k, and 32 kHz (Figure 5a). However, because the basal OHCs have lower levels of antioxidants and are more vulnerable to ROS damage [48,49], the increased thresholds shifts were more salient in the high frequency at 3 weeks after noise. 

Another possibility of circadian perturbation on acoustic trauma is the dysregulation of inflammatory responses [50,51]. Impaired circadian rhythm has been linked to pathological responses that are associated with the development of inflammatory and metabolic diseases [30,52]. In response to noise, a large influx of inflammation cells can be observed in the cochlea [53], causing upregulation of cochlear innate immunity [54] and proinflammatory molecules [55,56]. Since the chronic circadian disruption exaggerates the immune and inflammatory responses to external stimuli [57,58], it is reasonable to speculate that the dysregulation of the cochlear circadian clock may also increase the vulnerability of the cochlea to environmental stressors, such as noise. Particularly, glucocorticoids are well-known oscillators and are important in the modulation of auditory sensitivity to acoustic trauma [59,60]. LL affects the circadian rhythm of the hypothalamic–pituitary–adrenal (HPA) axis [61] and reduces the circadian amplitude of circulating corticosterone levels in C57BL/6J and Swiss Webster mice [34,57,62]. This may link the detrimental effect of LL in our study to increased noise-induced OHC loss, while glucocorticoids were thought as protective against acoustic trauma [60,63]. Of note, the LL-induced circadian plasma corticosterone change seems to vary among strains and animals [64], and the circadian effects of glucocorticoids and the receptors in CBA/CaJ mice are still unknown. Future investigation of the impact of circadian dysregulation on the molecular levels of proinflammatory cytokines and glucocorticoid receptors in the cochlea of CBA/CaJ mice after noise exposure will be helpful for our understanding of underlying mechanisms.

The results of our study could have important clinical implications. Physicians generally educate their patients to adjust their lifestyle to a regular sleep pattern. However, the habit of “to rise with the lark and go to bed with the lamb” is not easy in modern society since too many artificial illuminations from different sources, such as LED lamps and mobile devices (for example, cellphones and tablets), have deeply disrupted the circadian rhythm of the human body. Prolonged work hours throughout the evening, not uncommon in modern society, add to exposure to artificial light that can aggravate circadian disruption. Fortunately, circadian disturbance by LL does not affect the cochlear thresholds in the physiological state or with exposure to low-intensity noise. Our results, however, raise a red flag in regards to circadian dysregulation in excessively noisy environments, be it work or leisure, and emphasizes the need for adequate hearing protection. Alternatively, a compromised circadian clock can be re-adjusted by scheduled light exposure, feeding, or exercise [35], or pharmacological interventions such as melatonin [65]. As a note of caution, we have to consider that our model used LL to dysregulate the cochlear circadian clock, similar to the prolonged light environment in, for example, an intensive care unit. The use of animal models to mimic more common circadian-dysregulated situations such as shift work and jet lag [66] will be helpful in exploring the impact of circadian dysregulation on noise-induced hearing loss.

In summary, we observed that cochlear circadian dysregulation by LL leads to elevated PTS induced by high-intensity noise. Likewise, loss of OHCs and the reduction of synaptic ribbons were larger in the circadian-dysregulated mice exposed to high-intensity noise. In contrast, the deleterious effect of LL was not seen in TTS after low-intensity noise exposure. Our results suggest the importance of the normal circadian clock in the inner ear, particularly in the environment of high-intensity noise.

## 4. Materials and Methods

### 4.1. Animals

All animal research protocols and procedures were approved by the Institutional Animal Care and Use Committee, Kaohsiung Chang Gung Memorial Hospital (IACUC Affidavit #2015091901, approved 4 November 2015 and #2017121305, approved 15 March 2018). Male CBA/CaJ mice (4 weeks of age) were procured from National Laboratory Animal Center, Taiwan. Animals were provided with water and rodent chow at libitum. They were housed in an air-conditioned vivarium with 22 ± 1 °C and 12 h/12 h light–dark cycle (lights on: 5:00 (Zeitgeber time 0, ZT0); lights off: 17:00 (ZT12)) to acclimate for 2 weeks before the experiments. During lights-on, the light intensity was kept at about 270–320 lux (lm/m^2^).

### 4.2. Control and Alteration of the Light–Dark Cycle

Two weeks upon acclimating to the normal LD cycle, the mice were randomly divided into 3 groups. In the LD group, mice were housed in the 12 h/12 h LD cycle. In the DD group, animals were accommodated in a light-off condition. In the LL group, mice were housed in a light-on condition. The handling of mice held in the dark was performed under red light.

### 4.3. Noise Exposure

Three awake CBA/CaJ mice in the same group were placed in a sound chamber, with one mouse per stainless steel cage. The sound produced by a CD player (CD5001; Marantz, Kanagawa, Japan) was amplified using an amplifier (YS-150MP3; Yuan-Sin, Taiwan). The sound chamber was fitted with a loudspeaker (NSD 2005S-8; Eminence Speaker, LLC, Eminence, KY, USA). Broadband noise (BBN) with a frequency of 2 to 20 kHz was compiled onto a CD and equalized with software (CSL4400; Kay, NJ, USA). Before and after the noise exposure, sound levels were calibrated with a sound level meter (type 2250; Brüel & Kjær, Nærum, Denmark) fitted with a microphone (type 4189; Brüel & Kjær, Nærum, Denmark) at the locations of each cage within the sound chamber. For producing temporary or permanent threshold shifts, mice were exposed to 92 dB or 106 dB BBN noise for 2 h (from 8:00 am (ZT3) to 10:00 am (ZT5)). In the sham group, mice were placed in the sound chamber without noise.

### 4.4. Assessment of Auditory Brainstem Response (ABR) and Distortion Product Otoacoustic Emission (DPOAE)

Mice were anesthetized via an intramuscular injection of xylazine (Bayer) (10 mg/kg) and Zoletil 50 (Virbac) (25 mg/kg) and then placed on a heating pad to maintain their body temperature at around 37 °C. Acoustic stimuli were delivered monaurally to a Beyer earphone attached to a customized plastic speculum inserted in the ear canal. For the measurement of ABRs, electrodes were inserted at the subdermal vertex of the skull under the left and right ears (ground). ABRs were detected at 8, 16, and 32 kHz. Up to 1024 responses were averaged for each stimulus level. Thresholds were determined by decreasing the intensity in 10 dB increments, and then in 5 dB steps near the threshold until no responses were detected. Thresholds were estimated between the lowest stimulus level where a response was observed and the level without a response. ABR thresholds were tested before and 4 weeks after the LD cycle change in all mice (pre-noise ABR). Post-noise ABR was tested 3 h, 1 day, and 3 weeks after noise exposure in the temporary threshold shift experiment. Post-noise ABR was tested 1 day, 1 week, and 3 weeks after noise exposure in the permanent threshold shift experiment (see Figure 3). The ABR threshold shift was defined as the difference between the post- and pre-noise ABR thresholds. For the measurement of DPOAEs, the DPOAEs were generated by two simultaneous tones of frequencies F1 and F2, with F2/F1 ratio set as 1.2 and the F2 level 10 dB lower than the F1 level. The DPOAE input/output (I/O) function was plotted by the curve from the 2F1-F2 emission amplitudes at a fixed F2 frequency.

### 4.5. Real-Time Quantitative Polymerase Chain Reaction (qRT-PCR) Assay of mRNA Expression

Cochlear tissues were collected to assess the mRNA expression of circadian clock genes at 6 different ZTs or circadian times (CTs). Total RNA was isolated from cochlear tissues using the RNeasy Micro Kits (Qiagen, Valencia, CA, USA). Total RNA (2 µg) was reverse-transcribed to first-strand cDNA using High Capacity cDNA Reverse Transcription Kits (Applied Biosystems, Foster City, CA, USA) according to the manufacturer’s protocols. cDNA was diluted (1:10) with ddH_2_O and stored in aliquots at −20 °C. The expression of the circadian clock genes was analyzed using the TaqMan^®^ system. All TaqMan^®^ gene expression assays were purchased from Applied Biosystems. Expression of the mouse housekeeping gene, *Actb* (*β-actin*), was used to normalize expression of the circadian clock genes in qRT-PCR. All reactions were carried out in a 10 µL final volume containing 25 ng cDNA (as total input RNA), 0.5 µL 20× TaqMan^®^ Gene Expression Assay, and 5 µL 2× TaqMan^®^ Universal PCR Master Mix (Applied Biosystems). Real-time qPCR was performed on an ABI 7500 Fast Real-Time System (Applied Biosystems), and the PCR cycling parameters were set as follows: 95 °C for 10 min followed by 40 cycles of PCR reaction at 95 °C for 20 s and 60 °C for 1 min. The expressions of the circadian clock genes (*Per1*, *Per2*, *Rev-erbα*, *Bmal1*, and *Clock*) were normalized to the internal control *Actb* to obtain the relative threshold cycle (ΔCt). The relative expression levels were calculated by equalizing differences to the ΔCt of ZT0 of the LD group, so the expression level at ZT0 of the LD group equaled 100%. Each data point was the average of three technical replicates.

### 4.6. Surface Preparation of the Cochlear Sensory Epithelium

The temporal bones of the mice were removed immediately following euthanasia. Scala media was perfused with 4% paraformaldehyde in phosphate-buffered saline (PBS) and fixed at 4 °C overnight. The cochleae were rinsed in PBS and decalcified in 0.12 M sodium ethylenediaminetetraacetic acid (EDTA), which was changed daily for 2 days. Then the cochleae were further dissected by removing the softened otic capsule, stria vascularis, Reissner’s membrane, and tectorial membrane.

### 4.7. Immunocytochemistry for Outer Hair Cell (OHC) Counts

Upon incubating in 3% Triton X-100 in PBS for 30 min and washing 3 times with PBS, the specimens were incubated in rhodamine-phalloidin (1:100; Invitrogen, Carlsbad, CA, USA) for 60 min and followed by PBS rinses. The sections were mounted on glass slides with PermaFluor^TM^ Mountant (Thermo Fisher Scientific, Pittsburgh, PA, USA). Cell populations in the phalloidin-stained stereociliary bundles and circumferential F-actin rings around the cuticular plate of the OHCs were evaluated using an epifluorescence microscope with a 40× oil immersion objective lens. The right objective had a 0.17 mm calibrated scale imposed on the field for reference, and all three rows of OHCs were oriented longitudinally within each 0.17 mm frame. Each successive 0.17 mm field was evaluated beginning from the apex to the base for counting the number of OHCs. Cell counts were entered into a computer program (KHRI Cytocochleogram, version 3.0.7; Kresge Hearing Research Institute, University of Michigan, Ann Arbor, MI, USA) to calculate the percentage of OHC loss from the apical turn to the basal turn of the basal epithelium.

### 4.8. Immunocytochemistry for Synaptic Ribbon Counts and 4-Hydroxynonenal (4-HNE)

After blocking with 10% donkey anti-mouse serum (Abcam, Cambridge, UK) for 30 min at room temperature (22–24 °C), the basilar membrane was immunostained with primary mouse anti-C-terminal binding protein 2 (CTBP2) IgG1 monoclonal antibody at 1:200 dilution (BD Biosciences, San Diego, CA, USA) or primary rabbit anti-4-HNE polyclonal antibody at 1:50 dilution (Abcam, Inc., Cambridge, MA, USA), and incubated at 37 °C overnight or 4 °C for 72 h, which was co-labeled with donkey anti-mouse AlexaFluor 488-conjugated secondary antibody (Abcam) at 1:1000 dilution at 37 °C for 1 h in the dark. After washing three times with PBS, the tissues were incubated with rhodamine-phalloidin at 1:100 (Invitrogen) at room temperature for 60 min to visualize the structure. CTBP2 or 4-HNE immunostaining was observed by laser confocal microscopy (FV10i; Olympus, Tokyo, Japan) with a 60× magnification lens. For synaptic ribbon counts, regions of interest along the cochlear spiral at 5.6, 8, 16, 22.6, and 32 kHz were examined. The z-stack images were analyzed using the Image J software (NIH, Bethesda, MD, USA) “cell counter” plugin and the mean number of puncta per inner hair cell (IHC) base in the region of interest were counted. For quantification of immunolabeled 4-HNE signals, the immunostaining over each OHC in the basal segment of cochlea was measured. The background fluorescence intensity was subtracted and the average fluorescence of about 60 OHCs was calculated. The relative fluorescence was then quantified by normalizing the ratio of average fluorescence of OHCs in each condition to the LD (sham) group.

### 4.9. Immunohistochemistry for Cryosections

After the decalcification with 4% EDTA, cochleae were transferred to 30% sucrose and incubated overnight. The cochleae were embedded with OCT for sectioning. Sections of 7 µm thickness were permeabilized with 3% Triton X-100 in PBS for 30 min, washed 3 times with PBS and blocked with 10% donkey anti-mouse serum for 30 min at room temperature. The sections were then immunostained with primary rabbit anti-PER2 IgG1 polyclonal antibody at 1:100 (PER21-A, Alpha Diagnostic, San Antonio, TX, USA), and incubated at 4 °C overnight, which was co-labeled with donkey anti-mouse AlexaFluor 488-conjugated secondary antibody (Abcam) 1:1000 at 37 °C for 1 h in the dark. After washing 3 times with PBS, specimens were incubated with rhodamine-phalloidin (Invitrogen) at 1:100 and DAPI (Invitrogen) at 1:1000 at room temperature for 60 min to visualize the structure. The cryosections were then observed by laser confocal microscopy.

### 4.10. Statistical Analysis

GraphPad Prism 8.0 (GraphPad Software, La Jolla, CA, USA) was used for all the statistical analyses. One-way analysis of variance (ANOVA), two-way ANOVA, Tukey’s multiple comparisons, and unpaired *t*-tests were utilized to analyze the difference among groups. All tests were two-tailed, and a *p*-value < 0.05 was considered significant. CircWave 1.4 software (www.euclock.org) was used to calculate the waveform and to analyze the circadian rhythmicity of circadian clock gene expression in the LD, LL, and DD groups, using forward linear harmonic regression (F test). CircWave software fits one or more fundamental sinusoidal curves through the individual data points and compares this with a horizontal line through the data mean (a constant). If the fitted circadian clock gene expression pattern follows a sinusoidal curve and differs significantly from the horizontal line, it is considered circadian rhythmic (*p*-value < 0.05).

## Figures and Tables

**Table 1:** *p*-value of F test to detect the circadian rhythmicity of mRNA transcripts of circadian clock genes in the cochlea by CircWave software.

	LD Group	DD Group	LL Group
*Per1*	0.000012	0.023638	ns
*Per2*	<0.000001	0.001643	ns
*Rev-erbα*	<0.000001	0.008508	ns
*Bmal1*	0.000035	ns	ns
*Clock*	<0.000001	ns	ns

*p* < 0.05 indicates circadian rhythmicity, ns (not significant) means loss of circadian rhythm.

## Supplementary Materials

The following are available online at https://www.mdpi.com/1422-0067/21/20/7535/s1, Figure S1. Representative images of PER2 immunostaining in the cochlea and organ of Corti under normal LD cycle.

## Abbreviations

LD	light/dark
LL	constant light
DD	constant dark
ZT	Zeitgeber time
CT	circadian time
ABR	auditory brainstem response
DPOAE	distortion product otoacoustic emission
TTS	temporary threshold shift
PTS	permanent threshold shift
OHC	outer hair cell
IHC	inner hair cell
SCN	suprachiasmatic nucleus
BBN	broadband noise
qRT-PCR	real-time quantitative polymerase chain reaction
4-HNE	4-hydroxynonenal
