# Supplementary material for: Constant Light Dysregulates Cochlear Circadian Clock and Exacerbates Noise-Induced Hearing Loss
Source: Int J Mol Sci. 2020 Oct 13;21(20):7535. doi: 10.3390/ijms21207535 (PMC7589695; doi:10.3390/ijms21207535)
Supplement: Supplementary file 1 [file ijms-21-07535-s001.pdf]

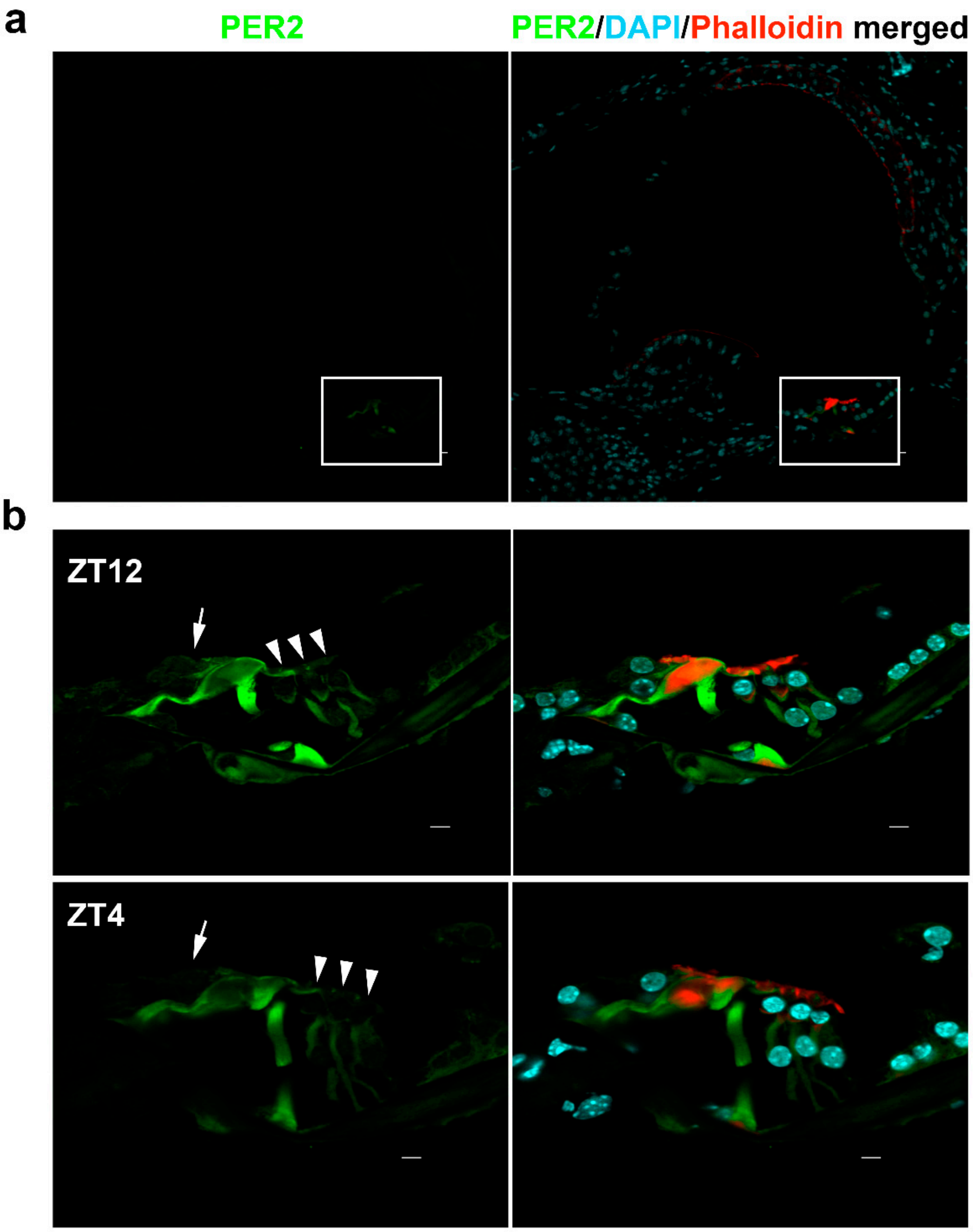

Supplementary Figure S1. Representative images of PER2 immunostaining in the cochlea and organ of Corti under normal LD cycle. **a.** Cochlear cryosections were immunostained for PER2 (green) (left panel) and counterstained with Rhodamine phalloidin to label F-actin (red) and DAPI to label nuclei (blue) (right panel). The rectangular box represents the organ of Corti. **b.** Images of the organ of Corti at ZT12 and ZT4. PER2 immunostaining was higher at ZT12 in the organ of Corti, in contrast to lower immunostaining at ZT4. Arrow indicates IHC. Arrowhead indicates OHCs. These images are representative of four individual mice from the LD group at ZT12 and ZT4. Scale bar = 5 μm.
